# Exonuclease processivity of archaeal replicative DNA polymerase in association with PCNA is expedited by mismatches in DNA

**DOI:** 10.1038/srep44582

**Published:** 2017-03-16

**Authors:** Takuya Yoda, Maiko Tanabe, Toshiyuki Tsuji, Takao Yoda, Sonoko Ishino, Tsuyoshi Shirai, Yoshizumi Ishino, Haruko Takeyama, Hirokazu Nishida

**Affiliations:** 1 Department of Life Science and Medical Bioscience, Waseda University, 2-2 Wakamatsu-cho, Shinjuku-ku, Tokyo 162-8480, Japan; 2 Hitachi, Ltd. Research & Development Group, 1-280 Higashi-koigakubo, Kokubunji, Tokyo 185-8601, Japan; 3 Department of Computer Bioscience, Nagahama Institute of Bio-Science and Technology, 1266 Tamura-cho, Nagahama, Shiga 526-0829, Japan; 4 Department of Bioscience and Biotechnology, Faculty of Agriculture, Kyushu University, 6-10-1 Hakozaki, Higashi-ku, Fukuoka, Fukuoka 812-8581, Japan

## Abstract

Family B DNA polymerases comprise polymerase and 3′ −>5′ exonuclease domains, and detect a mismatch in a newly synthesized strand to remove it in cooperation with Proliferating cell nuclear antigen (PCNA), which encircles the DNA to provide a molecular platform for efficient protein–protein and protein–DNA interactions during DNA replication and repair. Once the repair is completed, the enzyme must stop the exonucleolytic process and switch to the polymerase mode. However, the cue to stop the degradation is unclear. We constructed several PCNA mutants and found that the exonuclease reaction was enhanced in the mutants lacking the conserved basic patch, located on the inside surface of PCNA. These mutants may mimic the Pol/PCNA complex processing the mismatched DNA, in which PCNA cannot interact rigidly with the irregularly distributed phosphate groups outside the dsDNA. Indeed, the exonuclease reaction with the wild type PCNA was facilitated by mismatched DNA substrates. PCNA may suppress the exonuclease reaction after the removal of the mismatched nucleotide. PCNA seems to act as a “brake” that stops the exonuclease mode of the DNA polymerase after the removal of a mismatched nucleotide from the substrate DNA, for the prompt switch to the DNA polymerase mode.

Proliferating cell nuclear antigen (PCNA) is a ring-shaped protein that encircles DNA and plays numerous roles in nucleic-acid metabolism. It is an essential component of the DNA replication machinery and is required for DNA recombination and repair, as well as several other cellular processes. Particularly, PCNA performs the crucial function of providing replicative polymerases with the high processivity required to duplicate an entire genome^1^,^2^,^3^. Three PCNA monomers, each comprising two similar domains, are joined in a head-to-tail arrangement to form a homotrimer composed of six repeating domains^4^.

Many reports have described the recruitment of proteins to PCNA, which primarily interacts with binding proteins on the outside of the ring-shaped PCNA. These interactions are mediated by the conserved peptide motif called the ‘PCNA interacting protein (PIP) box’, which is widely observed in PCNA-binding proteins and hooked by the interdomain loop on the outside surface of PCNA^5^,^6^. Actually, the substitution of the interdomain loop of human PCNA with the corresponding region of yeast PCNA seriously affected the interaction with the human cell cycle protein, p21 7.

Structural studies have elucidated the interaction with DNA via the inside face of the PCNA ring^8^,^9^. Other reports have revealed the conformations of the complexes of DNA, PCNA, and DNA transaction enzymes^10^,^11^. Moreover, mutations of the basic amino acids on the inside face of the human PCNA ring decreased the synthetic activity of human DNA polymerase δ *in vitro*^12^. However, the distinct roles of the inside face of the PCNA ring in the other reactions during the DNA replication process, *e.g.* the exonucleolytic and DNA ligation reactions, remained to be clarified.

DNA replication in Archaea and Eukarya is considered to be performed mainly by family B DNA polymerases, composed of the polymerase and exonuclease domains responsible for the DNA synthesizing and editing (proofreading) reactions, respectively, although the *polB* gene can be deleted from some archaeal genomes and PolD probably functions in replication in those cases^13^,^14^. These replication enzymes exhibit full activity when complexed with PCNA. The replicating complex structure of a family B DNA polymerase revealed that the substrate DNA interacted solely with the polymerase domain, while the substrate DNA in the editing complex interacted exclusively with the exonuclease domain^15^,^16^,^17^.

We solved the crystal structure of *Pyrococcus furiosus* DNA polymerase BI (PfuPolB) complexed with the monomer mutant of the cognate PCNA, and constructed a functional model of the PfuPolB/PfuPCNA/DNA complex in the replicating mode. This structural analysis suggested a switching mechanism between the synthesizing and editing modes^18^. Combined with the succeeding high resolution EM analysis of the PfuPolB/PfuPCNA/DNA complex in the editing mode, we deduced the different PCNA/DNA interactions between the synthesizing and editing modes^19^.

In this study, we mutated the conserved residues on the inside of the PfuPCNA ring that may be responsible for the interactions with DNA, in both the synthesizing and editing modes. We assessed the binding affinities to DNA and the effects on the polymerase/exonuclease reactions, by comparing the wild type and mutant PCNAs. PfuPCNA can be loaded onto a circular DNA substrate without replication factor C (RFC) at 70 °C *in vitro*^20^. When linear DNA was employed as a substrate, PfuPCNA self-loaded onto DNA even at 37 °C, and stimulated DNA synthesis by human DNA polymerase δ *in vitro*^21^. Therefore, we assessed the PCNA-associated effect on the PfuPolB activities by self-loading reactions, in order to exclude the possible influences of the different loading efficiencies of the wild type and mutant PCNAs by RFC. The polymerase and exonuclease reactions revealed the distinct roles of the conserved basic amino acids that we focused on in this study. The basic amino acids seemed to play a key role in suppressing the exonuclease reaction when the mismatched nucleotides were removed from the dsDNA substrate. Moreover, we evaluated the ligation reactions by *P. furiosus* DNA ligase (PfuLig) with the wild type and mutant PCNAs. The stable interaction between DNA and PCNA facilitated the ligation reaction.

## Results

### The conserved basic residues on the inside face of PfuPCNA targeted for mutagenesis

**Figure 1:**
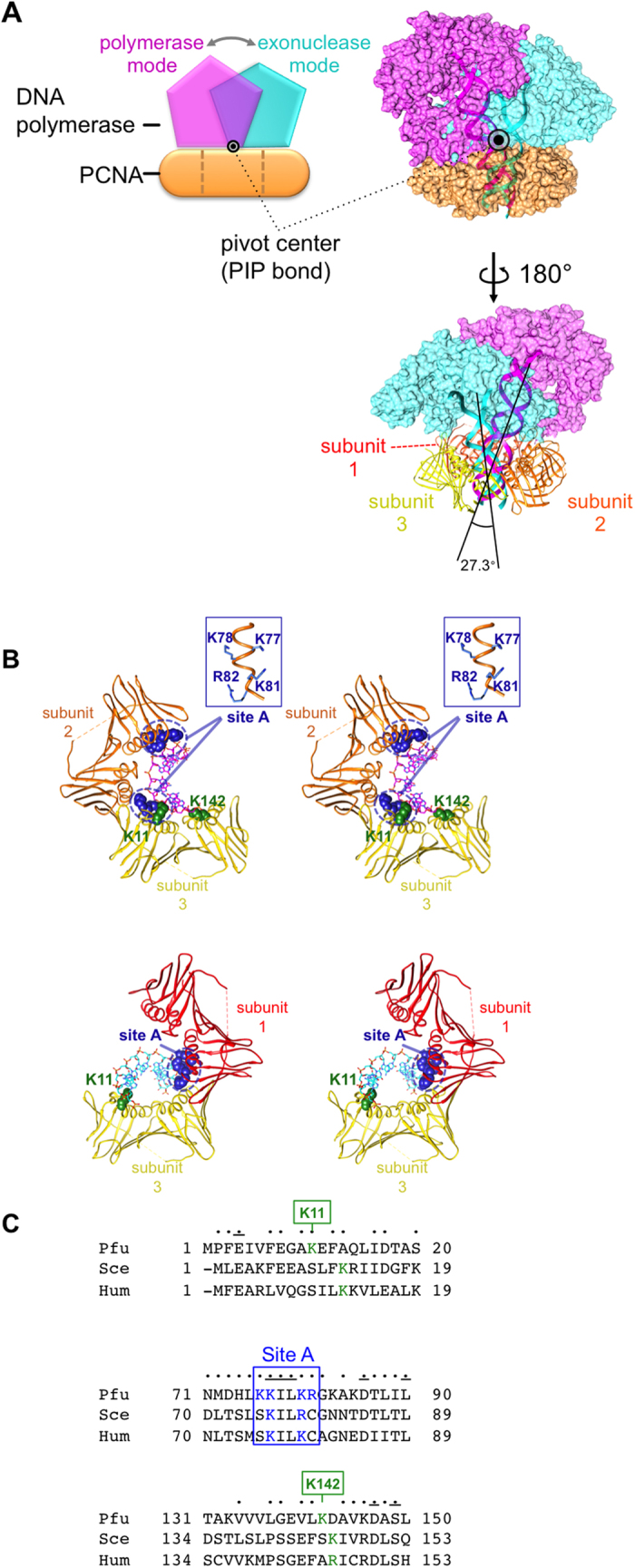
Models of the PfuPolB/PfuPCNA/DNA complex and the DNA interactions in the polymerase and exonuclease modes. (**A**) Schematic diagram of the pivot motion of PfuPolB on PfuPCNA between the polymerase-exonuclease modes, with the PIP bond (black circle) as the pivot center. PfuPolB in the polymerase mode is colored magenta, PfuPolB in the exonuclease mode is colored cyan, and PfuPCNA is colored orange. Ribbon representations of the substrate DNA molecules (magenta: polymerase mode, cyan: exonuclease mode) are superimposed on the surface model of the proteins. (**B**) Stereo views of the substrate DNA interactions with PfuPCNA in each mode. A stick model of the strand in elongation (magenta) is overlaid with the interacting subunits of PfuPCNA in the polymerase mode (top). Two sets of Site A (close-up view in the box) from subunits 2 and 3 interact with the DNA strand, flanked by Lys11 and Lys142. A stick model of the strand to be hydrolyzed (cyan) is superimposed on the relevant subunits of PfuPCNA in the exonuclease mode (bottom). (**C**) Amino acid alignment of the *Pyrococcus furiosus* (Pfu), *Saccharomyces cerevisiae* (Sce), and human (Hum) PCNAs. Amino acids conserved between two of the three organisms are marked by dots, and lines mark those conserved among all three organisms.

The structural studies of the PfuPolB/DNA/PfuPCNA complex revealed a substantial change between the polymerase and exonuclease modes (Fig. 1A, top)^16^,^17^,^18^,^19^. PfuPolB is tightly anchored on PCNA by the PIP motif, and rotates from the polymerase to exonuclease mode with the PIP motif as a pivot center (Fig. 1A, bottom).

The predicted models, combined with molecular dynamics analyses and experimentally determined DNA-bound sliding clamp structures, allowed us to estimate how the penetrating DNA substrates interact with PCNA in each mode^9^,^19^,^22^. In both modes, only the strand undergoing elongation (polymerase mode) or hydrolysis (exonuclease mode) interacts with the basic residue cluster of PCNA (designated as ‘Site A’), composed of Lys77, Lys78, Lys81, and Arg82. In addition, Lys11 and Lys142 are close to the phosphate group of the strand located at the exit of the ring (Fig. 1B). Note that the DNA substrate in the exonuclease model lacks a mismatch, and thus this model reflects the interactions after the removal of a mismatch.

We evaluated the conservation of Site A and some other basic residues among archaea and eukaryotes (Fig. 1C). The amino acid alignment of the *P. furiosus* (Pfu), *Saccharomyces cerevisiae* (Sce), and human (Hum) PCNAs revealed that the sequence around Site A (KILK(R)) is extensively conserved among the three organisms. In addition, the two basic residues (Lys11 and Lys142) that may participate in the interaction with DNA are also weakly conserved in the alignment.

We constructed a series of composite alanine-substituted mutants of the Site A and non-Site A residues. However, the substitution of an ionic residue of PfuPCNA sometimes seriously affected the homotrimer complex formation, and some of the alanine mutants of Site A failed to form the ring complex. As a result, we successfully purified the K78A, K81A, K77A/K78A, K78A/K81A, and non-Site A mutants of K11A, K142A and K11A/K142A in this study.

### SPR analyses of wild type and mutant PfuPCNAs on primed- or ds-DNA

SPR measurements were performed for the wild type and all mutants of PfuPCNA, using the 25 mer DNA primer on the 70 mer DNA template (p25/t70, primed-DNA) and the primer hybridized with the 25 mer DNA template (p25/t25, ds-DNA) as substrates. The kinetic parameters obtained from the SPR experiments are summarized in Tables S1 and S2. The *K*_D_ value for the wild type PfuPCNA with the primed-DNA substrate was considerably lower than the values for the single mutants of Site A (Table S1). The *K*_D_ values of the two composite mutants of Site A were markedly larger than that of the wild type, whereas the composite mutation of non-Site A exhibited weaker effects (Table S2). Although non-specific ionic interactions between PCNA and DNA might be partly responsible for these results, the difference in the *K*_D_ changes between Site A and non-Site A indicated that the Site A residues are essential for the interaction with DNA.

### SPR analyses of PfuPolB and PfuLig with wild type and mutant PfuPCNAs

To rule out the possibility that the mutated PfuPCNAs might have reduced affinity for PfuPolB and PfuLig, we performed the SPR experiments with PfuPolB and PfuLig, using the wild type or mutant (K78A/K81A) PfuPCNA immobilized substrate. The kinetic parameters are summarized in Table S3 and the representative sensorgrams are shown in Figure S1. The K_D_ values of PfuPolB obtained with the wild type and K78A/K81A were 8.036 × 10^−8^ and 7.466 × 10^−8^, respectively, and the K_D_ values of PfuLig with the wild type and K78A/K81A PfuPCNAs were 1.143 × 10^−6^ and 1.221 × 10^−6^, respectively. These results suggested that the mutations of PfuPCNA did not seem to affect the interactions between PfuPCNA and PfuPolB or PfuLig.

### SPR analyses of the complex of PfuPolB with wild type or mutant PfuPCNAs on primed-DNA

To assess the effect of the mutations of PfuPCNA on the interaction between the PfuPolB + PfuPCNA (the wild type or the mutant) complex and the primed DNA (p25/t70) substrate, we performed SPR experiments with PfuPolB mixed with the equimolar amount of the wild type or K78A/K81A PfuPCNA. The kinetic parameters are summarized in Table S4 and the representative sensorgrams are shown in Figure S2. The differences in the kinetic parameters were insignificant, as compared to the values obtained in the above SPR experiment between PfuPCNAs and the primed DNA, which reportedly differed by three orders of magnitude between the mutants and the wild type PfuPCNA. When the PfuPolB was added to the sample mixture, the PCNA trimeric ring seemed to be stabilized, and then the affinity to DNA was largely recovered.

### Primer extension reactions with/without wild type and mutant PfuPCNAs

Since the affinities of the mutant PfuPCNAs to the primed-DNA substrates are lower than that of the wild type, we incubated excess amounts of PfuPCNA (4-, 8-, and 16-fold excesses of PfuPCNA relative to the DNA substrate) with the DNA substrate, prior to starting the extension reaction by adding PfuPolB.

**Figure 2:**
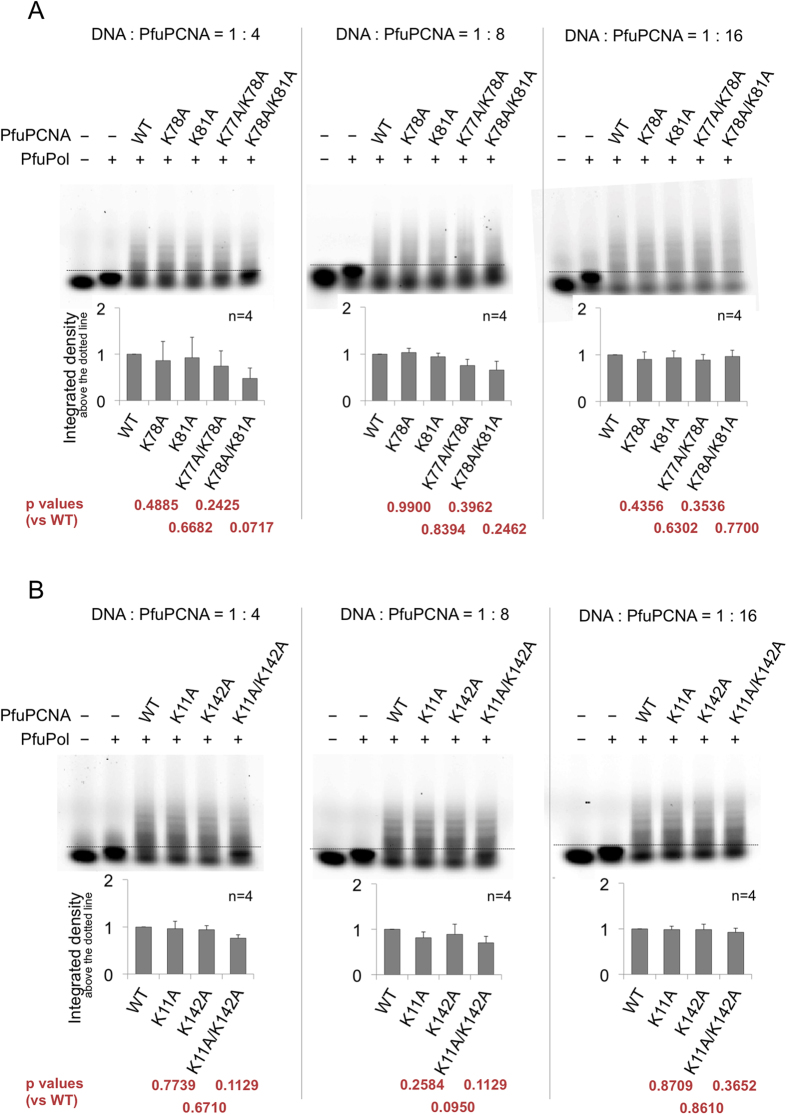
*In vitro* primer extension reactions with/without the wild type and mutant PfuPCNAs. The gel images displayed here are representative of several trials (n = 4). The bar charts at the bottom of the corresponding gel images show the average ratio of the quantities of the elongated primers to that of the unreacted primers and the non-PCNA-assisted-elongation band, as judged from the lane with PfuPolB alone, in which the ratio of the wild type was normalized to 1.0. The borders between the elongated and the unreacted (including non-PCNA assisted elongation) substrates are depicted as dotted lines. The ratios of the DNA substrates to the proteins (PfuPolB and PfuPCNA) in each panel are (**A**) 1:4, 1:8, and 1:16 (left to right), using the Site A mutants, and (**B**) 1:4, 1:8, and 1:16 (left to right), using the non-Site A mutants.

In the series of Site A mutants, the wild type is the most effective for the enhancement of the DNA elongation reaction, while the single mutations in Site A (K78A, K81A) showed slightly decreased polymerase enhancement, and the K78A/K81A Site A-knock out mutant exhibited extremely weak facilitation of DNA elongation, at a low ratio of PCNA to the DNA substrate (Fig. 2A, left). This may be caused by the weak interaction between PCNA and DNA. When a large excess of PCNA was added to the reaction mixture, the Site A-knock out mutants (K78A/K81A) showed comparable activity to the wild type (Fig. 2A, center and right). The results from the non-Site A mutants exhibited the same trend as in the case of the Site A mutants (Fig. 2B).

### Exonuclease reactions with/without wild type and mutant PfuPCNAs

**Figure 3:**
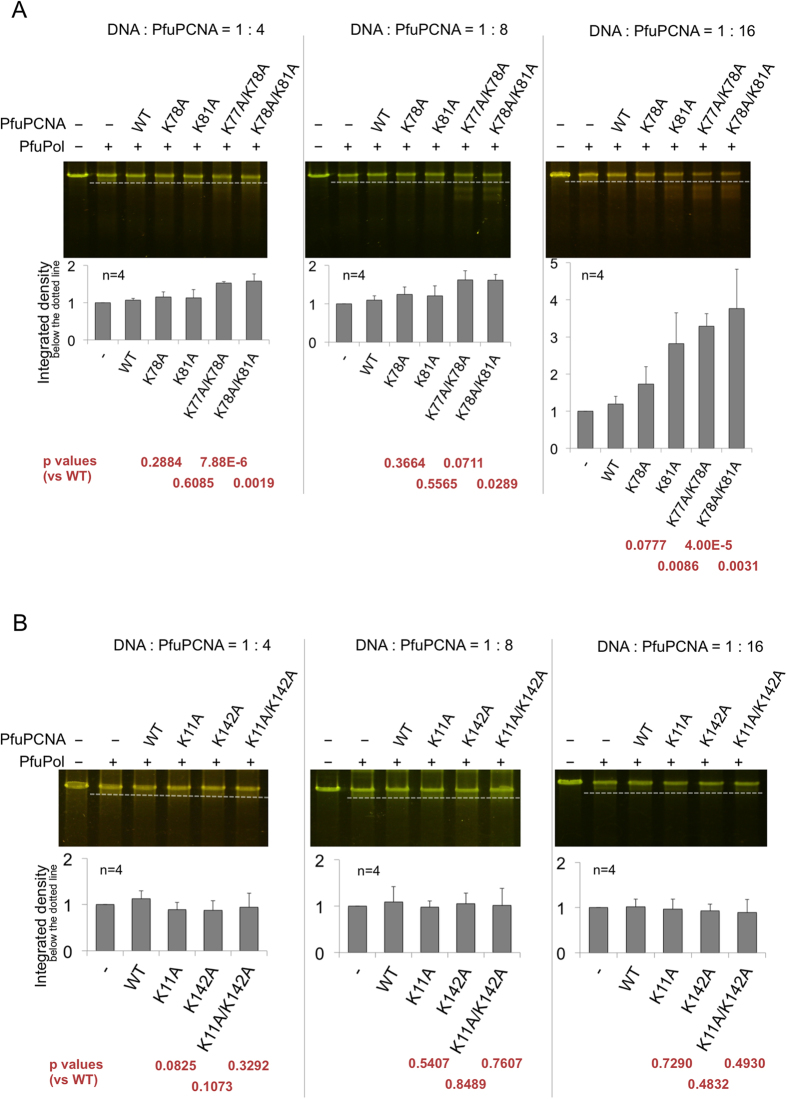
*In vitro* exonuclease reactions with/without the wild type and mutant PfuPCNAs. The gel images displayed here are representative of several trials (n = 4). The borders between the degraded and the unreacted (including non-PCNA assisted degradation) substrates are depicted as dotted lines. The ratios of the DNA substrates to the proteins (PfuPolB and PfuPCNA) in each panel are (**A**) 1:4, 1:8, and 1:16 (left to right), using the Site A mutants, and (**B**) 1:4, 1:8, and 1:16 (left to right), using the non-Site A mutants.

In contrast to the polymerase reaction, intriguingly, the mutant PCNAs considerably enhanced the exonuclease reaction, as compared to the wild type (Fig. 3). As the ratio of PCNA to the DNA substrate became higher, the facilitation by the Site A-knock out mutant (K78A/K81A) increased over those of the Site A-‘half’ knock out mutants, K78A and K81A, and thus the excess ratio of PCNA to the DNA substrate seemed to compensate for the low affinity of K78A/K81A for DNA, as observed in the polymerase reaction experiments (Fig. 3A, center and right). When K78A/K81A was mixed with PfuPolB, the affinity of the PfuPolB + PfuPCNA complex for DNA was not much lower than that of the wild type PfuPCNA complexed with PfuPolB (Table S4, Figure S2A). In contrast, in the case of PfuPCNA alone, the *K*_D_s differed by three orders of magnitude between the mutants and the wild type PfuPCNA. The K_D_ values of PfuPolB complexed with the wild type or K78A/K81A mutant were 4.783 × 10^−8 ^M and 8.864 × 10^−7 ^M, respectively, which might be compensated by the excess amount (*e.g.* × 16) of the K78A/K81A mutant. Indeed, increasing the amount of the added PfuPCNA resulted in the fading of the non-PCNA assisted degradation band (Figure S2B, highlighted in boxes), suggesting that the bands observed in the K78A/K81A lane in the 1:16 panel were mainly produced by the PfuPolB + PfuPCNA complex. In contrast, the non-Site A mutants, K11A and K142A, showed activities comparable to that of the wild type PfuPCNA (Fig. 3B).

In the cooperative reaction of PfuPCNA with PfuPolB in the exonuclease mode, PfuPCNA may simply encircle the DNA substrate, but not grip it. The coordination of the DNA substrate in the exonuclease reaction may be more flexible than that in the polymerase mode, in which the primer and template strands must be correctly located in the active site of DNA polymerase to confirm proper nucleotide pairing. In the polymerase mode, the energy for the DNA progression against the firm gripping by the two Site As, shown in Fig. 1B (top), must be provided by the high-energy phosphate bonds in the triphosphate substrates.

### Exonuclease reaction using dsDNA substrates with mismatches

**Figure 4:**
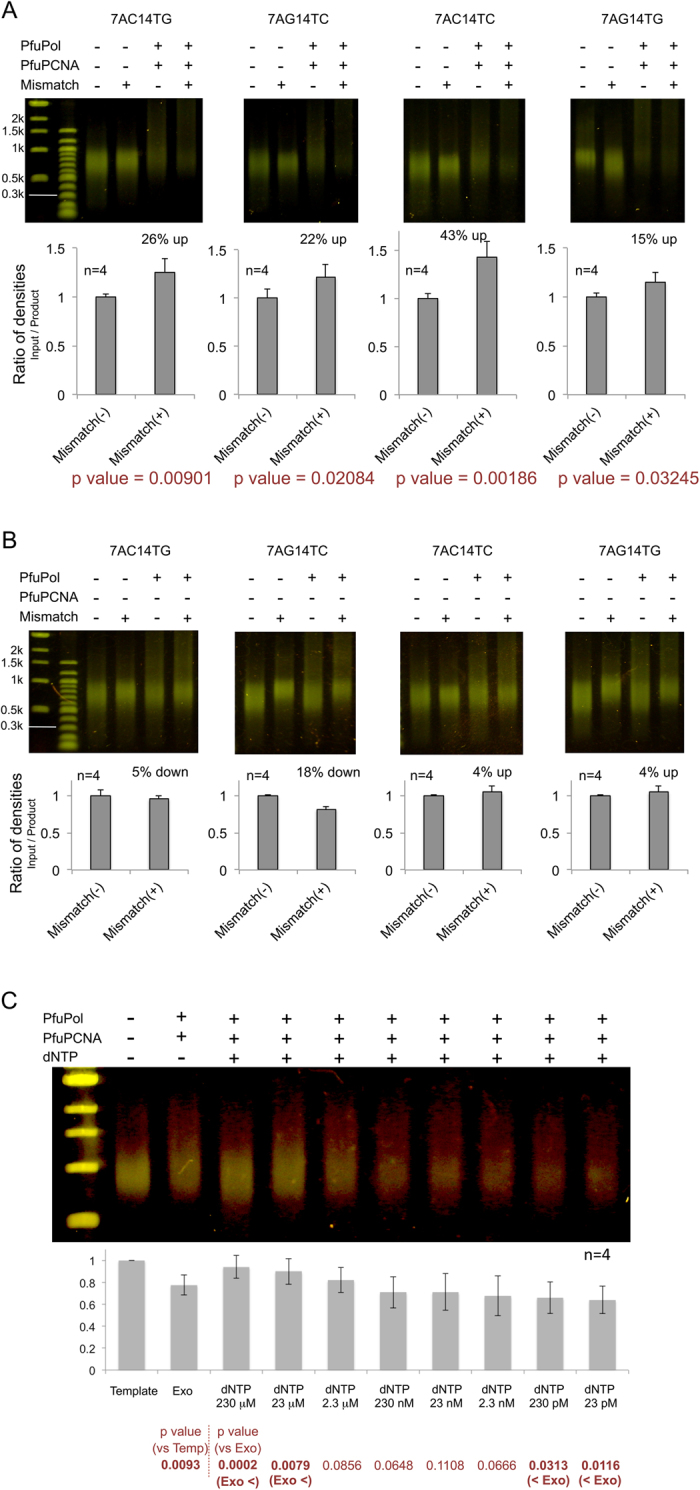
*In vitro* exonuclease reactions using the substrate dsDNA with/without several types of mismatches. The values of the degradation efficiencies using the substrates with (right) or without (left) mismatches are depicted in the bar charts, in which the values with the non-mismatched substrates were set to 1.0. (**A**) Exonuclease reactions with PCNA using the mismatch-induced substrates, compared to the substrate without a mismatch (left lane in each panel). The samples in the substrate only lanes (-PfuPolB, -PfuPCNA) were incubated in the same manner as those with enzymes. In this figure, the ratios of the densities were produced by dividing the density of the input template by that of the reaction product. (**B**) The same experiments without PCNA. (**C**) The addition of dNTPs (final concentrations of 230 μM, 23 μM, 2.3 μM, 230 nM, 23 nM, 2.3 nM, 230 pM, and 23 pM dNTPs) after the exonuclease reaction by PfuPolB + PfuPCNA using the mismatched substrate 4AG14TC, the high concentrations of dNTP (23 μM<) switches the enzyme from the exonuclease mode to the polymerase mode. On the other hand, at the lower concentration range of dNTP (<230 pM), the exonuclease reaction continued despite the existence of dNTP. The ratios of densities in the bar chart were produced by the dividing the density of the reaction product by that of the input substrate. The significant p values (<0.05) are in bold characters.

To assess the degree of the PfuPCNA-mediated enhancement in degradation reactions using DNA substrates with/without mismatches, we performed the exonuclease experiment using dsDNA substrates with several types of mismatches. Regardless of the type of mismatch, the exonuclease reaction on the mismatched DNA was facilitated by about 20%, as compared to the substrates without mismatches (Fig. 4A), whereas the exonuclease reaction without PCNA exhibited no enhancement for the mismatched substrates (Fig. 4B). The differences between the exonuclease activities with/without mismatches were statistically significant (Fig. 4A). This phenomenon was also observed with PolB from *Thermococcus kodakaraensis* (Figure S3). These results suggested that the mismatches in the substrates disrupted the interaction between Site A and the substrate DNA, predicted in the complex model depicted in Figure 1B (bottom)^19^, and then facilitated the degradation.

An additional experiment evaluated the critical dNTP concentration for the switching from the exonuclease to the polymerase modes (Fig. 4C). By this data, we demonstrated directly the critical dNTP concentration for the PfuPolB + PfuPCNA complex to switch from the exonuclease to the polymerase in our reaction condition *in vitro*.

No information about archaeal intracellular nucleotide concentration is available, but the average concentrations from about 600 published values in mammalian cells were approximately 5–37 μM dNTPs^23^.

Judging from the p values, in which the difference is considered to be significant if the values were lower than 0.05, an excess amount of dNTP equal to or higher than the physiological concentration (230 μM; p = 0.0002, 23 μM; p = 0.0079) seemed to force the PfuPolB + PfuPCNA complex into the polymerase mode even though the mismatches were included in the substrates. On the other hand, at a little lower dNTP concentration than the physiological condition (2.3 μM, p = 0.0856 > 0.05), the exonuclease and polymerase reactions counteract each other. The exonuclease reaction was not halted by the addition of dNTP, if the dNTP concentrations were much lower than the physiological condition (230 pM; p = 0.0313, 23 pM; p = 0.0116). The concentration of dNTP around the physiological concentration is the key for alternating the two modes.

### Potential energies of the complex in the exonuclease and polymerase modes

The potential energies of the PfuPolB/DNA/PfuPCNA complex in the exonuclease and polymerase modes exhibited large differences from the model-based calculations. The energy differences between the Site A mutants and the wild type PfuPCNA (*E*_mutant_ - *E*_wt_) in the exonuclease mode are 1,311, 1,087, 1,149, and 1,402 kcal/mol for K78A, K81A, K77A/K78A, and K78A/K81A, respectively. However, in the polymerase mode, the calculated energy differences are much smaller than those in the exonuclease mode: 86, −45, 365, and 253 kcal/mol for K78A, K81A, K77A/K78A, and K78A/K81A, respectively. These results are consistent with the observed effects of the mutations on the DNA extension and degradation activities, in which the degradation efficiency was seriously affected by the mutations.

### Nick-ligation reactions with/without wild type and mutant PfuPCNAs

**Figure 5:**
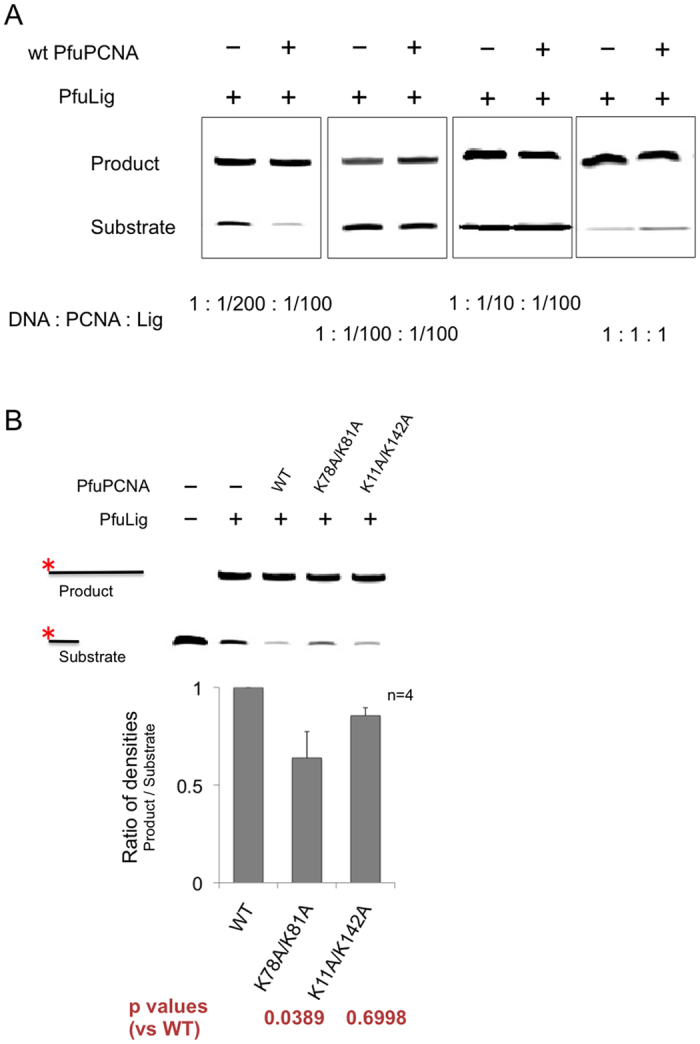
*In vitro* nick ligation reactions by PfuLig with/without the wild type and mutant PfuPCNAs. (**A**) Search for the conditions in which the addition of PfuPCNA enhances the nick ligation reaction by PfuLig. A large excess of the substrate DNA was required for the PCNA-enhanced nick ligation reaction. (**B**) *In vitro* nick ligation reactions of PfuLig with/without the wild type and mutant PfuPCNAs.

To evaluate the PfuPCNA-mediated facilitation of the nick-ligation reaction of PfuLig, we first searched for the appropriate concentrations of PfuPCNA, PfuLig, and nicked-DNA substrates (Fig. 5A)^24^. When the ratio of the substrate DNA to PfuPCNA/PfuLig was relatively low, no facilitation of the nick-ligation was observed even with the wild type PfuPCNA, whereas the facilitation effect by PfuPCNA was detected in the presence of a large excess of the substrate. Under the experimental conditions wherein the PCNA enhancement was observed, the wild type PfuPCNA was the most effective enhancer for the nick-ligation reaction by PfuLig, and the composite mutants of PfuPCNA (K78A/K81A and K11A/K142A) showed weaker facilitation of the reaction (Fig. 5B). The substrate should be tightly gripped by the DNA ligase without any motion during the ligation reaction, in contrast to the DNA polymerase reaction, which requires progressive motion along the DNA. Therefore, the rigid grip of PfuPCNA on the DNA substrate would enhance the DNA ligation reaction.

## Discussion

Since the atomic resolution structures of the Pol/PCNA/DNA complex in either the polymerase or exonuclease mode have not been solved, the basic amino acid cluster ‘Site A’ focused on in this study was discovered by analyses of the structural model of the ternary complex. This basic cluster is extensively conserved in the archaeal and eukaryotic PCNAs.

The dsDNA molecule is fully acidic, because its backbones consist of regularly repeated phosphate groups. The basic environment on the inside surface of the PCNA ring attracts dsDNA, and some of the positively charged amino acids are responsible for specific interactions with the phosphate groups of the substrate dsDNA. Site A seems to function as the specific support structure that guides the substrate dsDNA to the appropriate position within the PfuPolB active site.

The biochemical analyses of the PfuPolB activities in the polymerase and exonuclease modes, with/without the wild type or mutant PCNAs, revealed significant differences in the facilitation by PCNA. In the case of the polymerase reaction, when a low ratio of PfuPCNA to the DNA substrates was used, the Site A mutants of PfuPCNA produced only small amounts of the products extended by PfuPolB, as compared to the wild type (Fig. 2A, left and center). The reason for the lower amounts of the primer extension products is probably the weaker interaction between PCNA and DNA, as observed in the SPR experiments. When the substrate DNA was saturated with PfuPCNA, by the addition of a large excess of PfuPCNA, even the Site A-knock out mutants (K78A/K81A) exhibited activity comparable to the wild type (Fig. 2A, right) (in contrast to the SPR experiment, in which the binding affinities of the Site A mutants were quite low, the presence of PfuPolB might facilitate the efficient loading of PfuPCNA onto DNA), implying that the DNA recognition by the basic residues inside the PCNA ring does not affect the processing speed in primer extension. The weaker interaction between PCNA and DNA must diminish the friction hindering the progressive motion of Pol/PCNA along the DNA. However, at the same time, it may fail to correctly place the substrate in the active site of DNA polymerase, to confirm proper nucleotide pairing. Therefore, the facilitating and suppressing effects of the mutations seemed to compensate for each other to a certain extent in the polymerase reaction.

**Figure 6:**
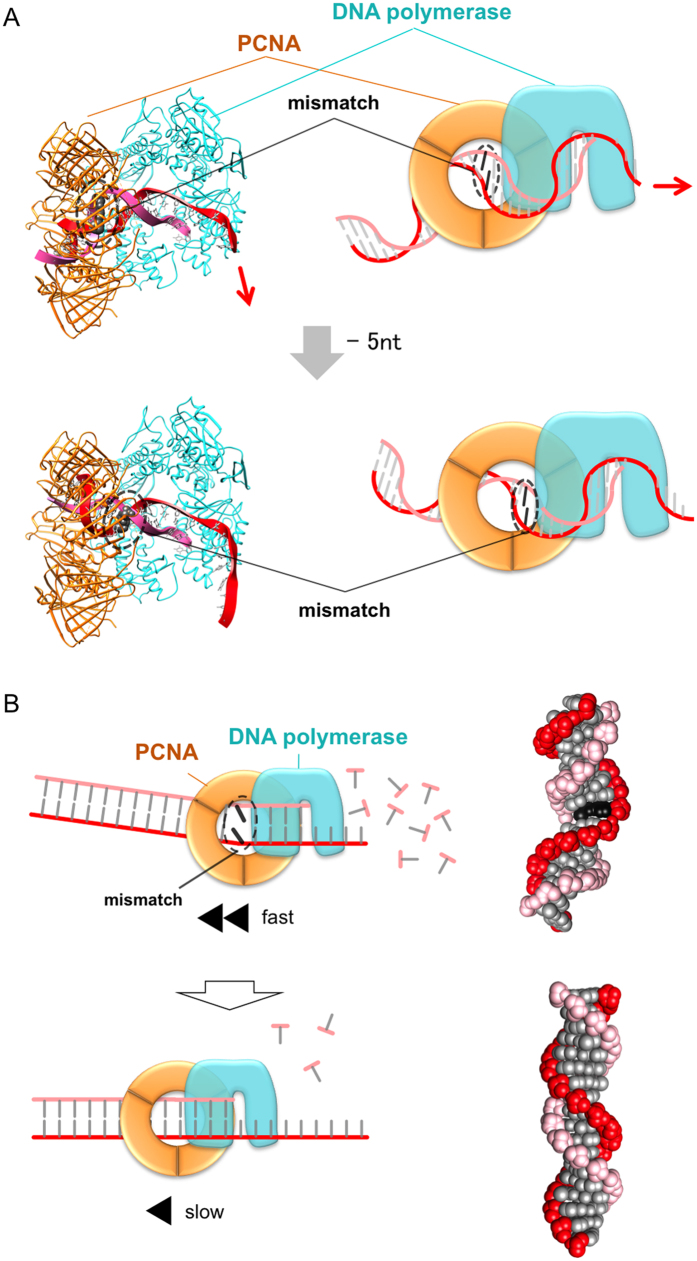
The difference in the stability of the interactions between mismatched- and ideal B-type double-stranded DNA. (**A**) Dynamic interaction between the substrate with a mismatch and the inside face of PCNA, in accordance with the progression of the exonuclease reaction. This figure was depicted by connecting the CA mismatch dsDNA with a substrate DNA binding to the PfuPolB/PfuPCNA complex in the exonuclease mode. On the one hand, if the location of the CA mismatch is the 11th position from the nucleolytic site, then the left portion of the substrate bends toward the lower side (top). On the other hand, if the location is the 6th position, then the substrate bends toward the upper side (bottom). Since the substrate is bending at the mismatched site, the latter portion of the substrate DNA undergoes a precession movement, and thus the DNA fails to interact stably with PCNA. Therefore, PCNA cannot brake the exonuclease reaction unless the mismatch is removed. (**B**) The difference in the exonuclease activities between mismatched- and ideal B-type double-stranded DNA. Schematic diagrams representing the difference in the efficacies of exonuclease reactions with a mismatched-dsDNA (top) or a correctly base paired-DNA (bottom), flanked by the tertiary structures of a mismatched-DNA in which the G-T mismatch pair is highlighted in black (top), and an ideal dsDNA (bottom).

In contrast, in the case of the exonuclease reaction, the basic amino acids in Site A appeared to inhibit the degradation of the correctly paired dsDNA substrates used in this study. The alanine-substitution mutations in Site A enhanced the nucleolytic degradation, suggesting that the specific ionic interaction between PfuPCNA and dsDNA is important for the nucleolytic reaction by itself. If the mismatch-containing dsDNA was used to form the ternary complex (PfuPolB/PfuPCNA/dsDNA), then the interactions between dsDNA and the amino acids in Site A should be disrupted. This would enhance the exonuclease reaction, since mismatched dsDNAs are prone to kink at their mispaired site, because of the lack of Watson-Crick interactions (Figure S4, top). Crystallographic analyses of the mismatch recognition protein MutS bound to mismatched dsDNA substrates revealed that the substrates were bent and recognized by MutS at their mispaired sites (Figure S4, bottom). The exonuclease reaction using the Site A mutant PCNAs might mimic the situation where a mismatched substrate is introduced into the PCNA ring. When a mismatch is located between PCNA and DNA polymerase, the portion following the mismatched site of the substrate undergoes a precession movement, changing the interaction between PCNA and DNA (Fig. 6A). After the removal of the mismatch, the continuous interaction with Site A, indicated in Fig. 1B (bottom), is restored. Thus, Site A should function as a “brake” that helps to stop the exonuclease reaction when the correctly paired dsDNA is formed in the PfuPolB/PfuPCNA binding region (Fig. 6B). Consistently, the exonuclease reactions with the mismatched substrates were facilitated, as compared to those using substrates without a mismatch. This result strongly supports the notion that PfuPCNA functions as a “brake” when the substrate without a mismatch binds between PfuPolB and PfuPCNA, as observed in the previous complex structure^19^. In contrast, when a mispaired site exists between PfuPolB and PfuPCNA, PfuPCNA cannot perform the “braking” task properly. In other words, when the flexible-mispaired site is located between PfuPolB and PfuPCNA, the substrate DNA might bend and interact weakly with PfuPCNA, resulting in the facilitation of the degradation reaction.

Furthermore, the energy differences of the complexes between the exonuclease and polymerase modes, which were much higher for the mutants in comparison to the wild type, support the notion that PCNA must constrain the exonuclease reaction when the normal dsDNA is introduced in the Pol/PCNA binding region, whereas PCNA does not curb the polymerase reaction with the normal dsDNA.

The biochemical analyses of the PfuLig activity with/without the wild type or mutant PfuPCNAs indicated that the mutation of the basic residues in PfuPCNA hindered the reaction, regardless of whether the mutation was in Site A or non-Site A. This is quite reasonable, since the DNA ligase reaction requires a tightly gripped DNA substrate^25^. Therefore, any substitutions of the basic residues located on the inside surface of the PCNA ring may decrease the efficiency of the PfuLig reaction.

In conclusion, Site A in PfuPCNA possibly functions as a “brake” that judges the appropriate time to stop the mismatch-degradation reaction, and seems to play a role as a “stabilizer” in the ligation reaction without successive motion along the DNA.

## Materials and Methods

### Site-directed mutagenesis of Lys11, Lys78, Lys81, Lys142, Lys77/Lys78, Lys78/Lys81, and Lys11/Lys142 to alanine

Polymerase chain reaction (PCR)–mediated mutagenesis was performed to change the AAG codons for Lys77, Lys78, and Lys81 to GCG. The plasmid pTPCNA (the structural gene encoding *Pyrococcus furiosus* PCNA (PfuPCNA) inserted into the pET21a vector (Novagen))^20^, was directly amplified by PCR using the following sets of primer pairs: K11Af (5′-ATTTG AAGGT GCAGC AGAGT TTGCC CAACTT-3′) and K11Ar (5′-AAGTT GGGCA AACTC TGCTG CACCT TCAAAT-3′), K78Af (5′-TGGAC CACCT AAAGG CGATC CTAAA GAGAG-3′) and K78Ar (5′-CTCTC TTTAG GATCG CCTTT AGGTG GTCCA-3′), K81Af (5′-AAGAA GATCC TAGCG AGAGG TAAAG CAAA-3′) and K81Ar (5′-TTTGC TTTAC CTCTC GCTAG GATCT TCTT-3′), K77A/K78Af (5′-TGGAC CACCT AGCGG CGATC CTAAA GAGAG-3′) and K77A/K78Ar (5′-CTCTC TTTAG GATCG CCGCT AGGTG GTCCA-3′), K78A/K81Af (5′-AAGGC GATCC TAGCG AGAGG TAAAG CAAA-3′) and K78A/K81Ar (5′-TTTGC TTTAC CTCTC GCTAG GATCG CCTT-3′), and K142Af (5′-GGAGA AGTCC TAGCA GATGC TGTTA AAGAT-3′) and K142Ar (5′-ATCTT TAACA GCATC TGCTA GGACT TCTCC-3′), which bind to the region containing the target codons (underlined). The PCR conditions involved 25 cycles of denaturation for 30 sec at 95 °C, annealing for 1 min at 55 °C, and extension for 8 min at 68 °C with Cloned *Pfu* DNA Polymerase (Agilent Technologies). The amplified plasmids were purified by WizardPlus SV Minipreps DNA Purification Systems (Promega) and used for transformation of *Escherichia coli (E. coli*) cells. The plasmids isolated from several independent transformants were sequenced. The plasmids that contained the gene with the mutation at the target codon were selected and utilized for protein expression.

### Purification of PfuPCNA, PfuPolB and PfuLig

The production and purification of the PfuPCNA mutants (K11A, K78A, K81A, K77A/K78A, K78A/K81A, K142A, and K11A/K142A) from *E. coli* BL21 (DE3) were performed as described previously for the wild type PfuPCNA^26^.

The procedures for the purification of *P. furiosus* DNA polymerase BI (PfuPolB) and DNA ligase (PfuLig) were performed as described previously, with slight modifications^27^,^28^.

### Surface plasmon resonance analyses

To examine the interactions of PfuPCNA and its mutants with DNA, a 5′-biotinylated 70 mer ssDNA (5′-TGCCG CCTCC AATTC TAATA CGACT CACTA TAGGG AGAAG GAAAC TCCAC CAACG ATCTG ACTAC TGCCT-3′) was bound to a Sensor Chip SA (GE Healthcare) coated with streptavidin, and then the complementary 25 mer ssDNA (5′-AGGCA GTAGT CAGAT CGTTG GTGGA-3′), designed to hybridize with the 3′- end of the 70 mer template, was applied to form the substrate primed-DNA (p25/t70). In addition, a 5′-biotinylated template 25 mer ssDNA (5′-TCCAC CAACG ATCTG ACTAC TGCCT-3′) with the complementary primer 25 mer ssDNA was used as the fully double-stranded dsDNA substrate (p25/t25). To measure the binding of PfuPCNA to the primed-DNA (p25/t70) or double-stranded-DNA (p25/t25), PfuPCNA was applied to the chip. To investigate the kinetic parameters, various concentrations (1.6, 0.8, 0.4, 0.2 μM) of the purified wild type, K11A, K78A, K81A, K77A/K78A, K78A/K81A, K142A, and K11A/K142A proteins were applied to the sensor chips.

To assess the interactions between the mutated PCNAs and PfuPolB or PfuLig, we performed the SPR experiment of PfuPolB and PfuLig using the wild type or K78A/K81A PCNA immobilized CM5 chips. To investigate the kinetic parameters, various concentrations (2, 1, 0.5, 0.25 μM) of PfuPolB or PfuLig were applied to the sensor chips.

We also performed the SPR experiment of PfuPolB mixed with the equimolar amount of the wild type or K78A/K81A PCNA. Various concentrations of PfuPolB + PCNA (2, 1, 0.5, 0.25 μM as a complex) were applied to the sensor chips. All measurements were performed at a continuous flow rate of 30 μl/min, in buffer containing 10 mM HEPES (pH 7.4), 150 mM NaCl, and 0.005% Tween 20. At the end of each cycle, the bound protein was removed by washing the chip with 4 M MgCl_2_.

### Construction of the DNA substrates and conditions for *in vitro* primer extension and exonuclease reactions

#### *In vitro* primer extension reaction

The DNA substrates for the primer extension reaction were prepared using M13mp18 single-stranded virion DNA (7,249 bases, Takara). A 20 mer ssDNA (5′-CTCTA GAGGA TCCCC GGGTA-3′) was hybridized to M13mp18 ssDNA for digestion by the restriction enzyme BamHI (Takara) for 1 h at 30 °C, resulting in the linear 7,249 ssDNA with the residual 12 mer (5′-GATCC CCGGG TA-3′) left at its 3′ terminus, in which the hybridized length was only 8 mer long (*T*_m_ = 28 °C). The residual primer was then easily replaced by an excess amount of the 30 mer primer for the extension experiment (5′-CCCGG GTACC GAGCT CGAAT TCGTA ATCAT-3′), by heating the solution at 95 °C for 2 min, followed by slow cooling.

The 20 μl reaction mixture, containing 20 mM Tris-HCl (pH 8.0), 100 mM NaCl, 2 mM MgSO_4_, 10 mM (NH_4_)_2_SO_4_, 50 mM KCl, 0.1% Triton X-100, 200 μM dNTP (containing 5 μM Cy5-labeled dCTP), 20 nM of template-primer DNA, and 40 (or 80, 160, 240) nM PfuPCNA or its mutants, was preheated at 55 °C for 4 min. The reaction was then started by adding 40 nM of DNA polymerase. After an incubation at 72 °C for 4 min, the reaction was stopped by adding 5 μl of stop solution, containing 98% deionized formamide, 1 mM EDTA, 0.1% xylene cyanol, and 0.1% bromophenol blue. The reaction products were fractionated by electrophoresis on an alkaline agarose gel (0.8%) containing 30 mM NaOH. The gels were analyzed with a FluoroImager 595 (GE Healthcare).

#### *In vitro* exonuclease reaction

The DNA substrates used in the exonuclease reaction were prepared as follows. The plasmid pTPCNA (the PfuPCNA structural gene inserted into the pET21a vector (Novagen), 6,193 bp, 24.5 nM) was solubilized in 100 μl of K buffer (Takara, 20 mM Tris-HCl, pH 8.5, 10 mM MgCl_2_, 1 mM dithiothreitol, 100 mM KCl) for digestion by 20 units of HindIII (Takara) at 37 °C for 12 hr, thus producing a linear 6,189 bp dsDNA with 4 mer (5′-TCGA-3′) ssDNAs on both 5′ ends. The HindIII-treated DNA solution was ethanol-precipitated, solubilized in dH_2_O, and adjusted to a 50 nM stock solution.

The 20 μl reaction mixture, containing 20 mM Tris-HCl (pH 8.0), 2 mM MgSO_4_, 10 mM (NH_4_)_2_SO_4_, 10 mM KCl, 0.1% Triton X-100, 0.1% nuclease-free BSA, 5 nM of the substrate DNA, and 10 (or 20, 40, 60) nM PfuPCNA or its mutants, was preheated at 55 °C for 4 min. The reaction was then started by adding a fixed amount (20 nM) of DNA polymerase. After an incubation at 72 °C for 30 min, the reaction was stopped by adding 5 μl of the stop reagent. After the addition of the stop solution, a 5 μl portion of the reaction solution was fractionated by 0.8% agarose gel electrophoresis at room temperature (100 V, 30 min) in TBE buffer (89 mM Tris/Tris-borate, pH 8.3, 2 mM EDTA). The gel was stained with 0.01% SYBR Gold (Life Sciences) in TBE for 30 min at room temperature. The gels were analyzed with a Safe Imager (Invitrogen).

#### *In vitro* exonuclease reaction with mismatched dsDNA

The DNA substrates used in the exonuclease reaction with mismatched dsDNA were prepared as follows. To construct the dsDNA substrates with mismatches at the underlined positions, the following primers were used: 5′- GGGG CGGCA GCAGT CAGGT CGTTG GTGGC -3′ (named 4G-p7AC14TG, in which “7AC” means that the 7th position from the 5′ end excluding the 4G sticky end was changed from A to C, and “14TG” means the 14th position was changed from T to G), 5′- GGGG CGGCA GGAGT CAGCT CGTTG GTGGC -3′ (4G-p7AG14TC), 5′- GGGG CGGCA GCAGT CAGCT CGTTG GTGGC -3′ (4G-p7AC14TC), and 5′- GGGG CGGCA GGAGT CAGGT CGTTG GTGGC -3′ (4G-p7AG14TG). In these primers, “5′-GGGG-” was utilized for the ligation between the units. These primers were used for the hybridization with the template 5′- CCCC GCCAC CAACG AACTG ACTAC TGCCG -3′ (4C-temp), in which “5′-CCCC-” was utilized for the ligation between the units.

We prepared another primer, 5′- GGGG CGGCA GTAGT CAGTT CGTTG GTGGC -3′ (4G-pNOMIS), for the creation of the dsDNA substrate without mismatches. When the primer 4G-pNOMIS hybridized with the template 4C-temp, there were no mismatches in the dsDNA substrate.

After the T4 polynucleotide kinase treatments, each primer-hybridized-template was incubated at 37 °C for 12 hr with T4 DNA ligase in the buffer supplied by the manufacturer (NEB). The enzyme-treated mixture was subjected to 1.0% agarose gel electrophoresis at room temperature (100 V, 30 min) in TBE buffer (89 mM Tris/Tris-borate, pH 8.3, 2 mM EDTA). The gel slices between approximately 0.8 to 1.2 kbp were excised, and the DNA was purified using a QIAquick Gel Extraction Kit (QIAGEN) for the 1 kbp substrate dsDNA. The molecular weight of the purified substrate was confirmed by electrophoresis on a 1.0% agarose gel.

The names of the resultant mismatch-including 1 kbp substrate dsDNAs are as follows: the names of the substrates made from the pairs of 4G-p7AC14TG and 4C-temp, 4G-p7AG14TC and 4C-temp, 4G-p7AC14TC and 4C-temp, and 4G-p7AG14TG and 4C-temp are “7AC14TG”, “7AG14TC”, “7AC14TC”, and “7AG14TG”, respectively.

The 20 μl reaction mixture, containing 20 mM Tris-HCl (pH 8.0), 2 mM MgSO_4_, 10 mM (NH_4_)_2_SO_4_, 10 mM KCl, 0.1% Triton X-100, 0.1% nuclease-free BSA, and 20 nM of the substrate DNA, with/without 160 nM PfuPCNA, was preheated at 55 °C for 2 min. The reaction was then started by adding a fixed amount (40 nM) of DNA polymerase. After an incubation at 60 °C for 10 min, the reaction was stopped by adding 5 μl of the stop reagent. The control substrate samples without PfuPolB and PfuPCNA were also processed in the same manner as those with enzymes. After the addition of the stop solution, a 12 μl portion of the reaction solution was fractionated by 1.0% agarose gel electrophoresis at room temperature (100 V, 30 min) in TBE buffer (89 mM Tris/Tris-borate, pH 8.3, 2 mM EDTA). The gel was stained with 0.01% SYBR Gold (Life Sciences) in TBE for 30 min at room temperature. The gels were analyzed with a Safe Imager (Invitrogen).

To compare the degrees of degradation in each case, the densities of each lane were integrated from 0.3 kDa to 2 kDa, using the ImageJ program. The integrated densities of the lanes processed by the enzymes were divided by that of the control lane without the enzymes. The resultant values correspond to the “degradation efficiency”. The values of the degradation efficiencies using the substrates with (right) or without (left) mismatches are depicted in the bar charts, in which the values with the non-mismatched substrates were set to 1.0.

#### Switching from exonuclease mode to polymerase mode by the addition of dNTPs

The 20 μl reaction mixture, containing 20 mM Tris-HCl (pH 8.0), 2 mM MgSO_4_, 10 mM (NH_4_)_4_SO_4_, 10 mM KCl, 0.1% Triton X-100, 0.1% nuclease-free BSA, and 20 nM of the substrate DNA (7AG14TC), with 160 nM PfuPCNA, was preheated at 55 °C for 2 min. The reaction was then started by adding a fixed amount (40 nM) of PfuPolB. After an incubation at 60 °C for 10 min, 2 μl of 2.5 mM, 250 μM, 25 μM, 2.5 μM, 250 nM, 25 nM, 2.5 nM, and 250 pM dNTPs were added to the reaction mixture in order to investigate the critical condition for the switching from the exonuclease mode to the polymerase mode. The reaction mixture was equally split into two tubes. In one tube, 5 μl of the stop reagent was added for estimating the exonuclease reaction. The other tube was incubated at 72 °C for 15 min for the polymerase reaction, and the reaction was stopped by adding 5 μl of the stop reagent. The control substrate samples without PfuPolB and PfuPCNA were also processed in the same manner as those with enzymes. After the addition of the stop solution, a 10 μl portion of the reaction solution was fractionated by 1.0% agarose gel electrophoresis at room temperature (100 V, 30 min) in TBE buffer (89 mM Tris/Tris-borate, pH 8.3, 2 mM EDTA). The gel was stained with 0.01% SYBR Gold (Life Sciences) in TBE for 30 min at room temperature. The gels were analyzed with a Safe Imager (Invitrogen).

### DNA ligation assay

The standard substrate used in the ligation assays was formed by annealing two short oligonucleotides, a 5′-phosphate-terminated 30 mer (5p-30) and a 5′-TET (tetrachloro derivative of carboxyfluorescein) labeled 20 mer (5TET-20), to a 40 mer complementary oligonucleotide target (T40). Their nucleotide sequences were as follows: T40, 5′-CAATC CTCTG GAGTC GACCT GTAGG AATGC AAGCT TGGCG-3′; 5p-30, Phosphate-5′-AGGTC GACTC CAGAG GATTG TTGAC CGGCC-3′; and 5TET-20, TET-5′-CGCCA AGCTT GCATT CCTAC-3′.

The nicked-DNA ligation activities of PfuLig with/without the wild type and mutant PfuPCNAs were measured using the above substrate. The reaction mixtures (15 μl) contained 0.01 mM ATP, 150 pmol 5′-TET labeled nicked-DNA substrate, 1.5 pmol of PfuLig, 0.75 pmol of the wild type or mutant PfuPCNA, and buffer B (20 mM Tris-HCl (pH 7.5), 20 mM KCl, 10 mM MgCl_2_, 0.1% Igepal, 1 mM DTT). The reactions were initiated by the addition of the enzymes, and were halted by the addition of a stop reagent (20 mM EDTA and 90% formamide). The samples were heated at 95 °C for 5 min and then electrophoresed through an 11% polyacrylamide gel containing 8 M urea in TBE (89 mM Tris/Tris-borate; 2 mM EDTA)^24^,^29^. The quantities of the products in the ligation reaction were estimated from the fluorescent band intensities on the gel, obtained with a FluoroImager 595 (GE Healthcare).

### Molecular dynamics simulation and potential energy calculations

Potential energy calculations were performed based on the snapshot structures obtained by molecular dynamics (MD) simulations at 300 K. We first added residues that were missing in the initial model structure, using Modeller (ver. 9.13)^30^. After a short (500 steps) potential energy minimization with position restraints of non-hydrogen atoms, TIP3P water molecules and counter ions (Na^+^) were added to neutralize the system. The numbers of added water molecules were 75,329 and 69,145 for the exonuclease mode and polymerase mode models^31^, respectively. We then performed a potential energy minimization (500 steps), a 50 ps NVT MD simulation, and a 100-ps NPT MD simulation, restraining non-hydrogen atoms to their initial positions. The ff14sb potential-energy function was used for the solute molecules^32^. We used the GPU version of the pmemd module of the AMBER software package (ver. 14) for all MD calculations^33^. The temperature was maintained at 300 K with the Langevin thermostat, with the collision frequency *γ* = 5 ps^−1^. In the NPT simulations, the pressure was maintained at 1.0 bar by using the Berendsen barostat, with the relaxation time *τ* = 2 ps^34^. The bonds involving hydrogens were constrained by SHAKE^35^. The time step for integration was 2 fs. Electrostatic interactions were evaluated by PME^36^.

We performed a 310 ns NPT MD simulation without position restraints, in which the final model structures were used for energy calculations. The side chains in the final models were changed to prepare four mutant (K78A, K81A, K77A/K78A, and K78A/K81A) models of the polymerase mode and the exonuclease mode. Potential-energy minimization of the wild type and mutant models was performed by the steepest descent method followed by the conjugate gradient method, until the root mean square gradient of the potential energy was < 0.1 kcal/mol/Å^2^, with the Molecular Operating Environment (MOE) program (Chemical Computing Group, Montreal, Canada) using an AMBER12 force field. The potential energies of the minimized models were calculated with the MOE program.

## Additional Information

**How to cite this article:** Yoda, T. *et al*. Exonuclease processivity of archaeal replicative DNA polymerase in association with PCNA is expedited by mismatches in DNA. *Sci. Rep.* **7**, 44582; doi: 10.1038/srep44582 (2017).

**Publisher's note:** Springer Nature remains neutral with regard to jurisdictional claims in published maps and institutional affiliations.

## Supplementary Material

Supplementary Figures and Tables
