# Supplementary material for: Exonuclease processivity of archaeal replicative DNA polymerase in association with PCNA is expedited by mismatches in DNA
Source: Sci Rep. 2017 Mar 16;7:44582. doi: 10.1038/srep44582 (PMC5353730; doi:10.1038/srep44582)
Supplement: Supplementary Figures and Tables [file srep44582-s1.doc]

Supplementary Information

Exonuclease processivity of archaeal replicative DNA polymerase in association with PCNA is expedited by mismatches in DNA

Takuya Yoda, Maiko Tanabe, Toshiyuki Tsuji, Takao Yoda, Sonoko Ishino, Tsuyoshi Shirai, Yoshizumi Ishino, Haruko Takeyama, and Hirokazu Nishida

**Table S1**

Kinetic parameters obtained from the SPR experiment with the wild type and Site A mutant PCNAs using the primed DNA (p25/t70) substrate (*A*), and the double stranded DNA (p25/t25) substrate (*B*).

A

| PCNA | p25/t70 primed DNA | | |
| --- | --- | --- | --- |
|  | *k*_a_ [M^-1^s^-1^] | *k*_d_ [s^-1^] | *K*_D_ [M] |
| WT | 8.160±0.590 × 10^4^ | 5.749±0.370 × 10^-3^ | 7.046 × 10^-8^ |
| K78A | 1.339±0.084 × 10^5^ | 1.451±0.046 × 10^-2^ | 1.084 × 10^-6^ |
| K81A | 1.762±0.330 × 10^3^ | 6.759±0.380 × 10^-3^ | 3.835 × 10^-6^ |
| K77A/K78A | 8.846±0.280 × 10^2^ | 5.115±0.130 × 10^-2^ | 5.783 × 10^-5^ |
| K78A/K81A | 2.644±0.150 × 10^2^ | 4.161±0.150 × 10^-2^ | 1.574 × 10^-4^ |

| PCNA | p25/t25 ds DNA | | |
| --- | --- | --- | --- |
|  | *k*_a_ [M^-1^s^-1^] | *k*_d_ [s^-1^] | *K*_D_ [M] |
| WT | 5.284±0.470 × 10^4^ | 5.628±0.410 × 10^-3^ | 1.065 × 10^-7^ |
| K78A | 1.212±0.098 × 10^4^ | 2.111±0.057 × 10^-2^ | 1.742 × 10^-6^ |
| K81A | 2.130±0.430 × 10^2^ | 9.210±0.096 × 10^-3^ | 4.325 × 10^-5^ |
| K77A/K78A | 4.229±0.770 × 10^2^ | 1.167±0.160 × 10^-2^ | 2.761 × 10^-5^ |
| K78A/K81A | 3.542±0.230 × 10^2^ | 7.436±0.390 × 10^-2^ | 2.099 × 10^-4^ |

**B**

**Table S2**

Kinetic parameters obtained from the SPR experiment with the wild type and non-Site A mutant PCNAs using the primed DNA (p25/t70) substrate (*A*), and the double stranded DNA (p25/t25) substrate (*B*).

A

| PCNA | p25/t25 ds DNA | | |
| --- | --- | --- | --- |
|  | *k*_a_ [M^-1^s^-1^] | *k*_d_ [s^-1^] | *K*_D_ [M] |
| WT | 7.191±0.110 × 10^4^ | 6.897±0.190 × 10^-3^ | 9.591 × 10^-8^ |
| K11A | 1.068±0.080 × 10^5^ | 1.243±0.042 × 10^-1^ | 1.164 × 10^-6^ |
| K142A | 1.861±0.055 × 10^5^ | 1.929±0.027 × 10^-1^ | 1.037 × 10^-6^ |
| K11A/K142A | 2.267±0.079 × 10^4^ | 4.105±0.064 × 10^-1^ | 1.811 × 10^-5^ |

**B**

| PCNA | p25/t70 primed DNA | | |
| --- | --- | --- | --- |
|  | *k*_a_ [M^-1^s^-1^] | *k*_d_ [s^-1^] | *K*_D_ [M] |
| WT | 1.077±0.017 × 10^5^ | 6.820±0.170 × 10^-3^ | 6.332 × 10^-8^ |
| K11A | 1.201±0.065 × 10^5^ | 6.726±0.180 × 10^-2^ | 5.600 × 10^-7^ |
| K142A | 2.145±0.110 × 10^5^ | 1.094±0.056 × 10^-1^ | 5.100 × 10^-7^ |
| K11A/K142A | 2.148±0.070 × 10^4^ | 1.602±0.150 × 10^-1^ | 7.458 × 10^-6^ |

**Table S3**

Kinetic parameters obtained from the SPR experiment with PfuPolB and PfuLig using the wild type or K78A/K81A PCNA immobilized substrate.

Table S4

| Analyte  Protein | Substrate  PCNA | Parameters | | | |
| --- | --- | --- | --- | --- | --- |
|  | | *k*_a_ [M^-1^s^-1^] | | *k*_d_ [s^-1^] | *K*_D_ [M] |
| PfuPolB | WT | | 1.058±0.043 × 10^5^ | 8.500±0.290 × 10^-3^ | 8.036 × 10^-8^ |
|  | K78A/K81A | | 1.243±0.050 × 10^5^ | 9.280±0.280 × 10^-3^ | 7.466 × 10^-8^ |
| PfuLig | WT | | 4.591±0.120 × 10^3^ | 5.246±0.110 × 10^-3^ | 1.143 × 10^-6^ |
|  | K78A/K81A | | 4.397±0.220 × 10^3^ | 5.367±0.110 × 10^-3^ | 1.221 × 10^-6^ |

Kinetic parameters obtained from the SPR experiment with PfuPolB complexed with the wild type or K78A/K81A PCNA using the primed DNA (p25/t70) substrate.

| Protein | p25/t70 primed DNA | | |
| --- | --- | --- | --- |
|  | *k*_a_ [M^-1^s^-1^] | *k*_d_ [s^-1^] | *K*_D_ [M] |
| PfuPolB +  WT PCNA | 1.075±0.017 × 10^5^ | 5.089±0.100 × 10^-3^ | 4.733 × 10^-8^ |
| PfuPolB +  K78A/K81A PCNA | 1.970±0.093 × 10^4^ | 1.746±0.040 × 10^-2^ | 8.864 × 10^-7^ |


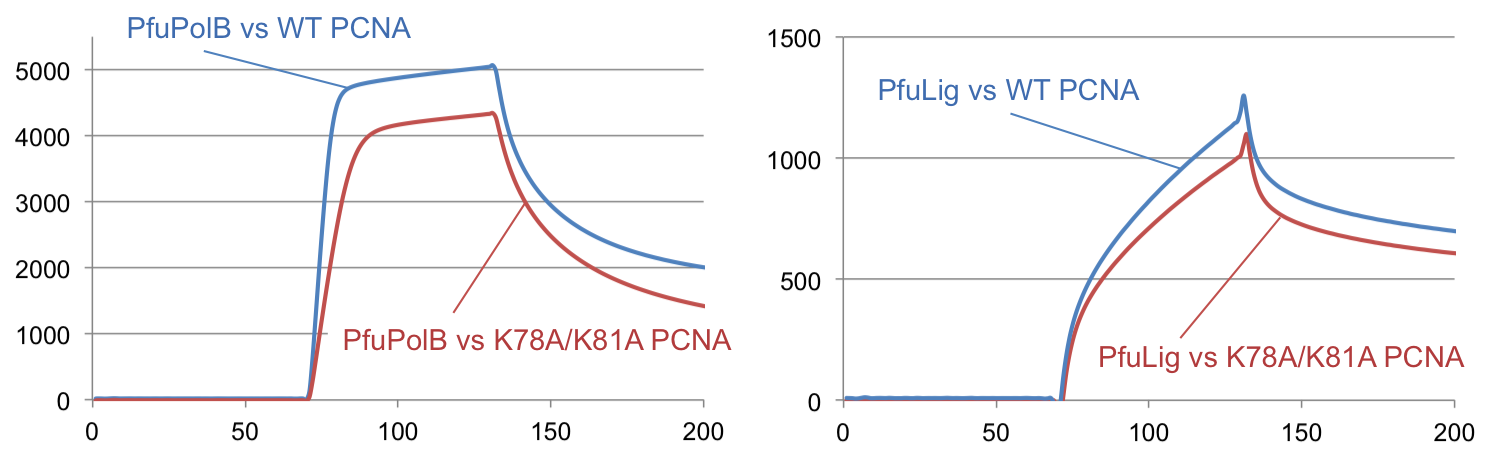


Fig. S1


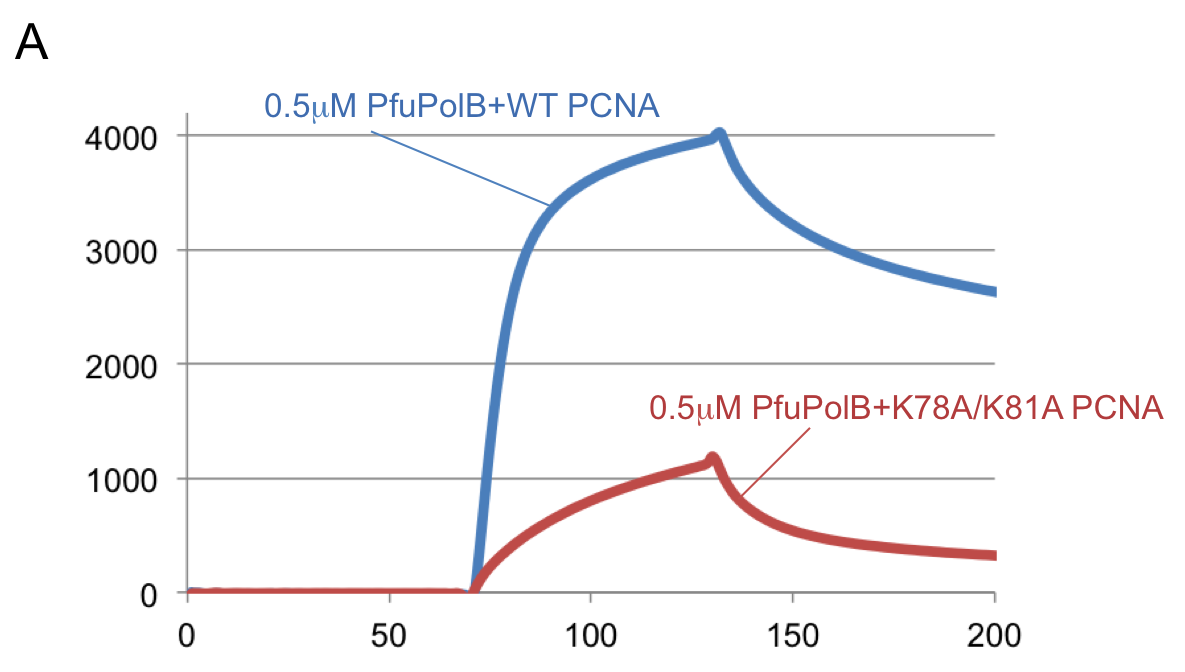


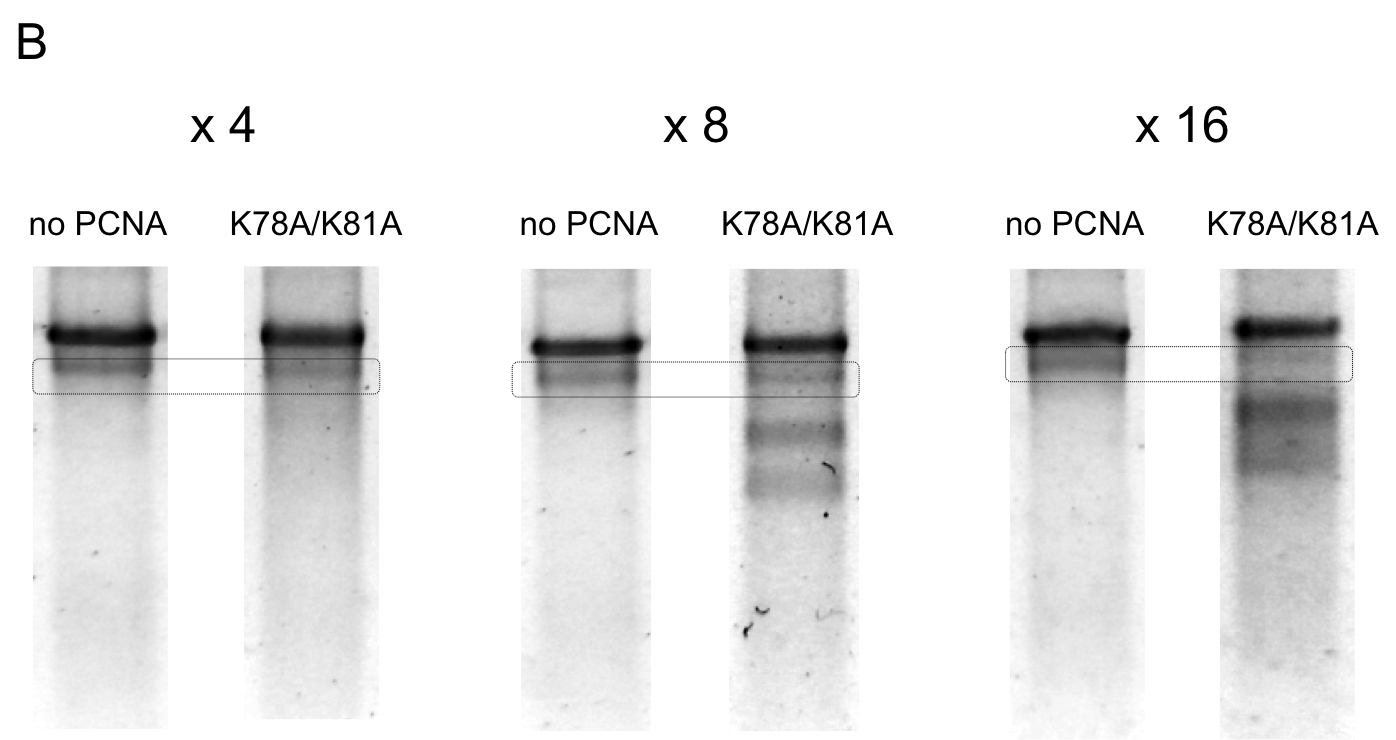


Fig. S2


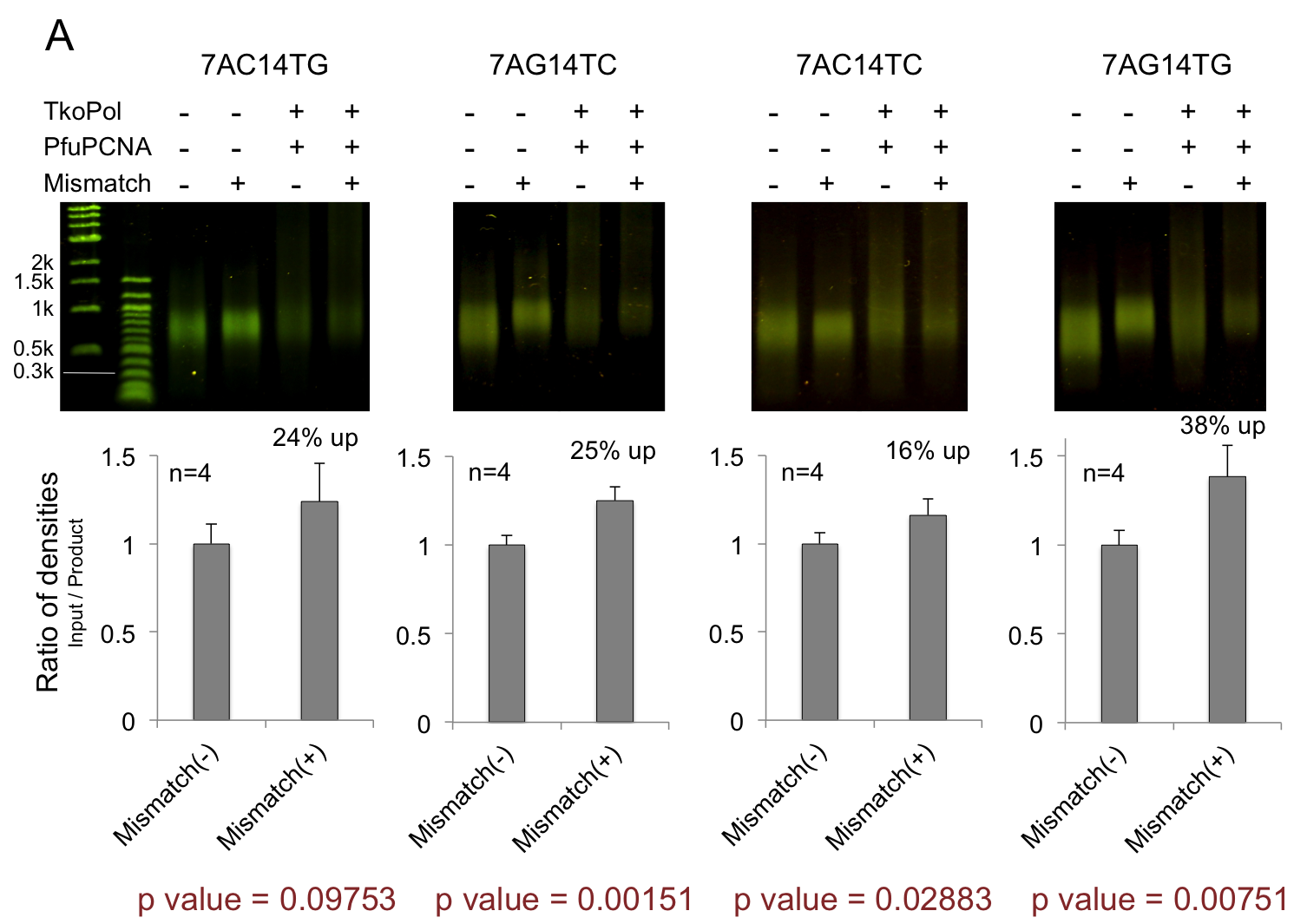

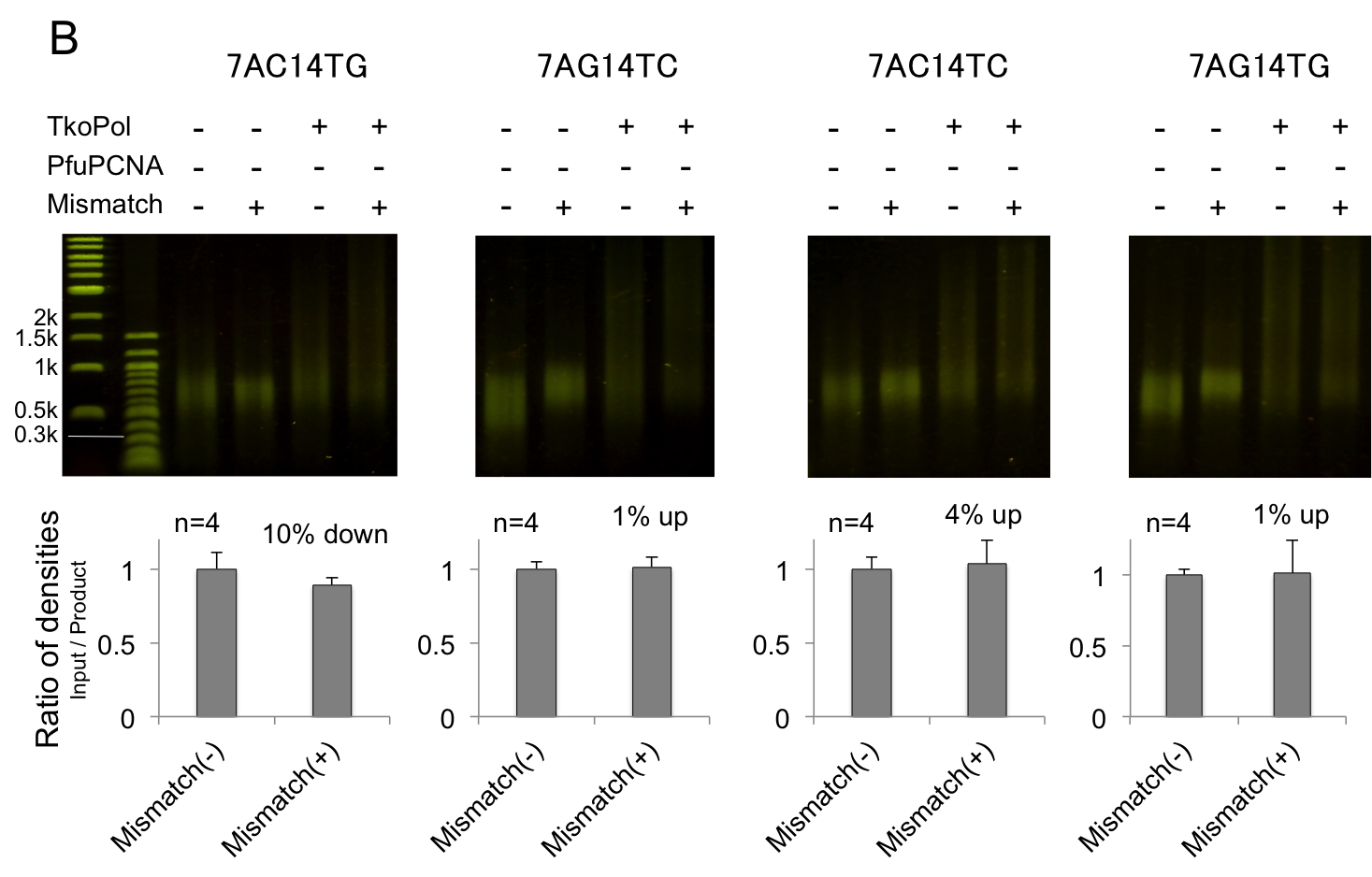


Fig. S3


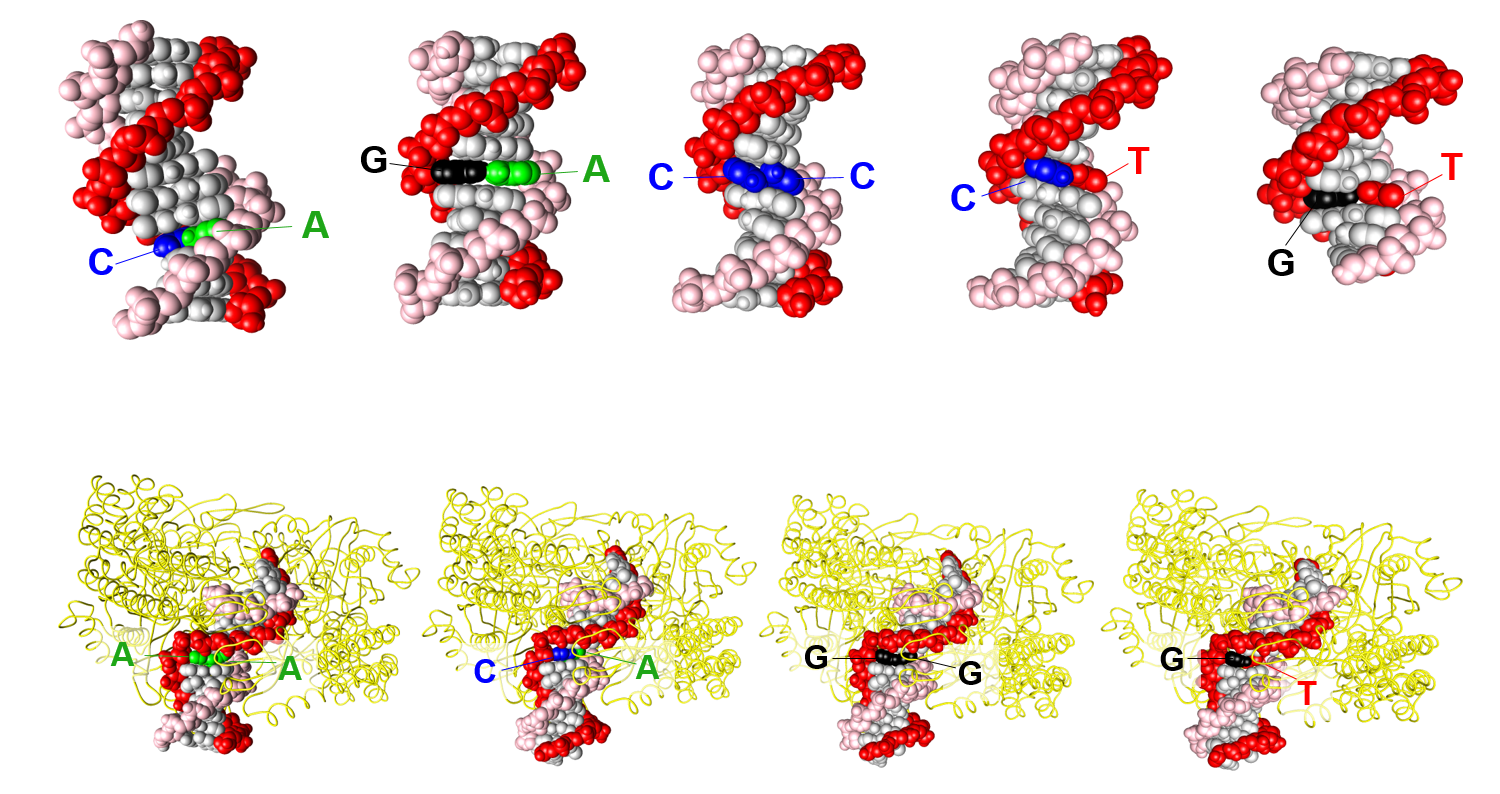


Fig. S4

Supporting Figure Legends

Fig. S1

SPR analyses of PfuPolB (0.5 μM, left) and PfuLig (2 μM, right) using the wild type PCNA (left) or the K78A/K81A mutant (right) immobilized CM5 chip.

Fig. S2

Characteristics of the PfuPolB+PfuPCNA complex in solution. (*A*) SPR analyses of PfuPolB complexed with the wild type or K78A/K81A mutant PfuPCNA, using the p25/t70 primed DNA immobilized streptavidin (SA) chip. When the 1:1 premixed PfuPolB and PfuPCNA solution was added to the primed DNA solution, a stoichiometric (1:1:1) complex of PfuPolB+PfuPCNA+DNA was obtained (1). (*B*) Close up view of the gel images of the exonuclease reaction from Figure 3A. Bands observed in the dotted rectangles correspond to the non-PCNA assisted degradation. This band is observed in the no PCNA lanes regardless of the amount of the added enzymes. In contrast, the band in the PfuPolB+K78A/K81A lane faded away according to the increase of the added enzymes, indicating that the low affinity of K78A/K81A for the DNA was compensated by the increased amount of the enzymes.

Fig. S3

*In vitro* exonuclease reactions with substrate dsDNAs with/without several types of mismatches, using DNA polymerase from *Thermococcus kodakaraensis* (TkoPolB) with PfuPCNA. The values of the degradation efficiencies using the substrates with (right) or without (left) mismatches are depicted in the bar charts, in which the values with the non-mismatched substrates were set to 1.0. (*A*) Exonuclease reactions with PCNA using the mismatch-induced substrates, compared to the substrate without a mismatch (left lane in each panel). The samples in the substrate only lanes (-TkoPolB, -PfuPCNA) were incubated in the same manner as those with enzymes. (*B*) The same experiments without PCNA.

Fig. S4

Structures of the mismatched DNA. The solution structures of the mismatched DNA determined by the nuclear magnetic resonance method (top) (2-4), and the crystal structures of the mismatched dsDNA in complex with the mismatch recognition protein MutS from *E. coli* (bottom) (5-8). The solution structures were first superimposed on each other, and then aligned in parallel. Nucleotides related to the mispairing in these dsDNAs are colored green (A: adenine), blue (C: cytosine), black (G: guanine), and red (T: thymine).

REFERENCES

1. Nishida H, Matsumiya S, Tsuchiya D, Ishino Y, Morikawa K (2006) Stoichiometric complex formation by proliferating cell nuclear antigen (PCNA) and its interacting protein: purification and crystallization of the DNA polymerase and PCNA monomer mutant complex from *Pyrococcus furiosus*. *Acta Cryst.* F62: 253-256.

2. Sanchez AM, Volk DE, Gorenstein DG, Lloyd RS (2003) Initiation of repair of A/G mismatches is modulated by sequence context. *DNA Repair* 2(8): 863-878.

3. Boulard Y, Cognet JAH, Fazakerley GV (1997) Solution structure as a function of pH of two central mismatches, C**-**T and C**-**C, in the 29 to 39 K-ras gene sequence, by nuclear magnetic resonance and molecular dynamics. *J Mol Biol* 268(2): 331-347.

4. Isaacs RJ, Rayens WS, Spielmann HP (2002) Structural differences in the NOE-derived structure of G-T mismatched DNA relative to normal DNA are correlated with differences in ^13^C relaxation-based internal dynamics. *J Mol Biol* 319(1): 191-207.

5. Natrajan G, et al. (2003) Structures of *Escherichia coli* DNA mismatch repair enzyme MutS in complex with different mismatches: a common recognition mode for diverse substrates. *Nucl Acids Res* 31(16): 4814-4821.

6. Lebbink JH, et al. (2010) Magnesium coordination controls the molecular switch function of DNA mismatch repair protein MutS. *J Biol Chem* 285(17): 13131-13141.

7. Junop MS, Obmolova G, Rausch K, Hwieh P, Yang W (2001) Composite active site of an ABC ATPase: MutS uses ATP to verify mismatch recognition and authorize DNA repair. *Mol Cell* 7(1): 1-12.

8. Alani E, et al. (2003) Crystal structure and biochemical analysis of the MutS**-**ADP**-**beryllium fluoride complex suggests a conserved mechanism for ATP interactions in mismatch repair. *J Biol Chem* 278(18): 16088-16094.
